# Endoscopic ultrasound–directed transenteric endoscopic retrograde cholangiopancreatography for biliary interventions in altered anatomy: tips and techniques

**DOI:** 10.1016/j.vgie.2026.04.045

**Published:** 2026-05-06

**Authors:** Michael Lajin, Kevin Bui, Tyler Jackson, Michael Khanjyan

**Affiliations:** Sharp HealthCare, San Diego, California, USA

## Abstract

**Background and Aims:**

Traditionally, deep enteroscopy, surgery, or percutaneous biliary drains were used to gain access for biliary interventions in patients with Roux-en-Y reconstruction. These methods are cumbersome, less successful than standard ERCP, and carry a higher risk of adverse events (AEs). Transductal and transmural EUS-assisted techniques are increasingly used in these settings. Although EUS-directed transgastric ERCP is mainly used for Roux-en-Y gastric bypass, EUS-directed transenteric ERCP (EDEE) is used for Roux-en-Y hepaticojejunostomy (RYHJ) and Roux-en-Y gastrectomy.

**Methods:**

In this case series, we presented 3 patients who underwent EDEE for stones, strictures, or both. We highlighted the technical differences between RYHJ and Roux-en-Y gastrectomy, as well as the different methods to distend the targeted small-bowel loop.

**Results:**

The biliary orifice was easily reached, and biliary interventions were successfully performed in all patients using the necessary accessories. There were no AEs directly related to the procedures.

**Conclusions:**

EDEE is an important technique for biliary interventions in patients with nongastric bypass Roux-en-Y reconstruction. Further data are needed to assess its long-term effectiveness and safety.

## Background and aims

Roux-en-Y reconstruction poses a challenge for endoscopic biliary interventions because it separates the alimentary limb (where the endoscope is inserted) from the biliary limb that leads to the biliary tree (the target for intervention).

Traditionally, biliary interventions assisted by enteroscopy and percutaneous methods have been used to avoid surgery.

In enteroscopy-assisted ERCP, an enteroscope is inserted into the alimentary limb, guided to the biliary limb at the jejunojejunal anastomosis, and then advanced to the biliary orifice.

This poses 2 main challenges: (1) an access challenge (difficulty reaching the biliary orifice) and (2) an execution challenge (caused by using a nondedicated endoscope, resulting in difficult cannulation, unstable positioning, and the inability to use certain tools).

These procedures often require a longer time with the patient under anesthesia and have a higher risk of failure than conventional ERCP.^1^^,^^2^

Percutaneous transhepatic biliary drainage (PTBD) allows biliary decompression and provides access for tools such as cholangioscopy and electrohydraulic lithotripsy (EHL). However, it carries the inherent risks of percutaneous access, such as bleeding, as well as risks associated with the drain, including dislodgment, obstruction, and leakage. It can be cumbersome for patients and may adversely affect their quality of life.

EUS-guided techniques are increasingly used to address these challenges. These procedures establish access to the biliary tree through one of the following platforms: (1) a transmural ductal access by connecting the alimentary limb directly to the biliary duct; these procedures are also referred to as EUS-guided antegrade treatment^3^; (2) EUS-guided gastroenteric or enteroenteric anastomosis by connecting the alimentary limb to the biliary limb to facilitate accessing the biliary orifice. This approach is further classified by the target organ: (a) In gastric bypass anatomy, the alimentary limb (gastric pouch/proximal jejunum) is connected to the target organ (the excluded stomach), and the procedure is called EUS-directed transgastric ERCP^4^; (b) in other Roux-en-Y anatomies, such as Roux-en-Y hepaticojejunostomy (RYHJ) and Roux-en-Y gastrectomy, the alimentary limb (stomach, duodenum, jejunum) is connected to the target organ (small bowel), and the procedure is called EUS-directed transenteric ERCP (EDEE).^5^, ^6^, ^7^, ^8^, ^9^

Although EUS-guided antegrade treatment techniques can still present access challenges, such as reaching a pathology in a right-sided intrahepatic duct (IHD) via EUS-guided hepaticogastrostomy, EUS-guided anastomosis methods address both access and execution challenges. By creating a short route, they facilitate access to the biliary orifice with a dedicated duodenoscope, enabling cannulation and the use of necessary accessory devices, and allowing repeated simple reintervention with shorter anesthesia exposure to the patient. In addition, these procedures enable an echoendoscope to access the biliary orifice, potentially offering further diagnostic^10^^,^^11^ and therapeutic advantages.

## Technical principles and tips

There are 2 technical variants for EDEE (Video 1, available online at www.videogie.org): (1) in RYHJ, the alimentary limb (naïve antrum/duodenum) is connected (during EDEE) to the biliary limb near the hepaticojejunostomy (HJ); (2) in Roux-en-Y gastrectomy, the alimentary limb (stomach/jejunum) is connected (during EDEE) to the biliary limb near the ligament of Treitz.

Multiple methods are available to identify and distend the targeted small bowel: (1) if the patient has a pre-existing PTBD, the drain is replaced with an 8F catheter without side holes, connected to an auxiliary pump for saline and contrast instillation; (2) if the targeted loop is visible (easier in RYHJ with the echoendoscope positioned in the antrum or the duodenal bulb), it is punctured directly with a 19-gauge needle connected to an auxiliary pump; (3) otherwise, an EUS-guided transhepatic approach can be performed by puncturing a left IHD with a 19-gauge needle and injecting about 50 to 60 mL of contrast mixed with saline solution to opacify the targeted small-bowel loop. Once identified, it can be further distended using a direct needle puncture. This method requires prompt drainage of the injected biliary tree either through a 1-step EDEE, an EUS-hepaticogastrostomy, or a temporary PTBD. As a result, this procedure needs to be scheduled earlier in the day, after coordination with interventional radiology (IR); (4) if EA-ERCP fails but the enteroscope reaches the biliary limb, a catheter-based approach can be used to facilitate EDEE.

## Patients and methods

### Patient 1

An 87-year-old woman with a history of atrial fibrillation and a GI stromal tumor resection with distal gastrectomy and Roux-en-Y reconstruction presented with elevated liver enzymes and abdominal pain. MRCP revealed a dilated bile duct filled with large stones (up to 22 mm).

On CT, a good apposition was observed between a blind jejunal loop beyond the surgical gastrojejunostomy and the proximal jejunum at the ligament of Treitz (Fig. 1).

**Figure 1:**
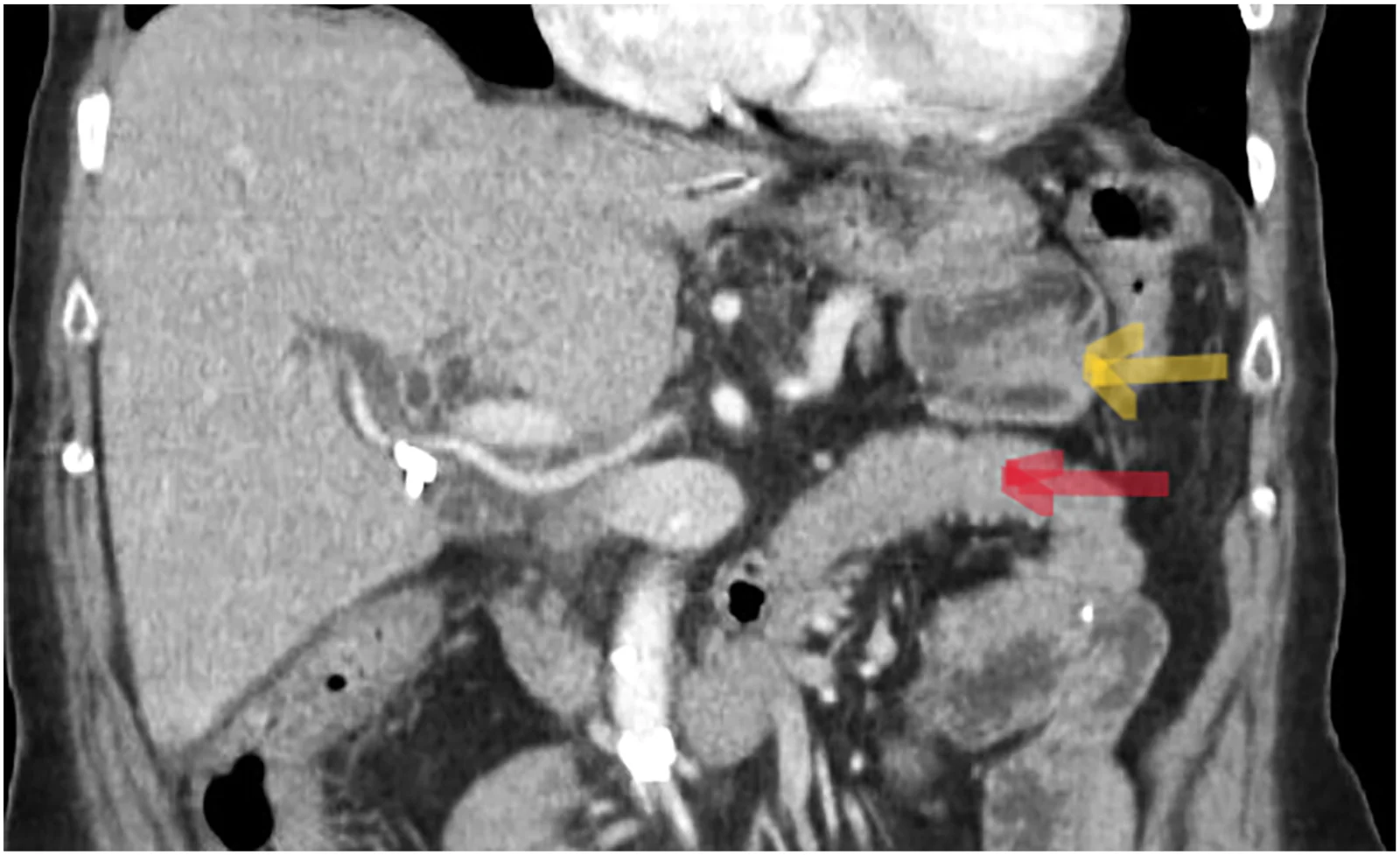
Patient 1: preoperative CT demonstrating a good apposition between the blind jejunal loop (*yellow arrow*) from the alimentary limb and the jejunum at the ligament of Treitz (*red arrow*).

After a multidisciplinary discussion with the patient and the IR and surgical teams, a decision was made to proceed with EDEE, considering the high likelihood of requiring cholangioscopy with EHL.

We discussed with our IR team the potential need for percutaneous biliary access if the targeted small-bowel loop was not visualized on EUS, and they agreed to be on standby.

A gastroscope was advanced into the blind jejunal loop, where a wire was left in place. A linear echoendoscope was then advanced to the blind jejunal loop. The targeted small-bowel loop could not be identified on EUS.

After administering antibiotics and glucagon, we used a 19-gauge needle to puncture a left IHD from the stomach. Contrast was injected into the biliary tree, opacifying the most proximal jejunal loop, which was punctured with the needle for further distention using an auxiliary pump. After adequate distension, a 2- × 1-cm electrocautery-enhanced lumen-apposing metal stent (EC-LAMS) (AXIOS, Boston Scientific, Marlborough, Mass, USA) was successfully deployed using electrocautery (Autotome settings [Olympus, Tokyo, Japan]).

Because of angulation at the lumen-apposing metal stent (LAMS) entry, ERCP was deferred until track maturation to prevent stent dislodgement. EUS-guided hepaticogastrostomy was not performed because of an intervening staple line. The IR team placed a PTBD shortly afterward to prevent cholangitis.

Postoperative CT confirmed an enteroenterostomy at the intended site, creating a short retrograde route to the papilla (Fig. 2).

**Figure 2:**
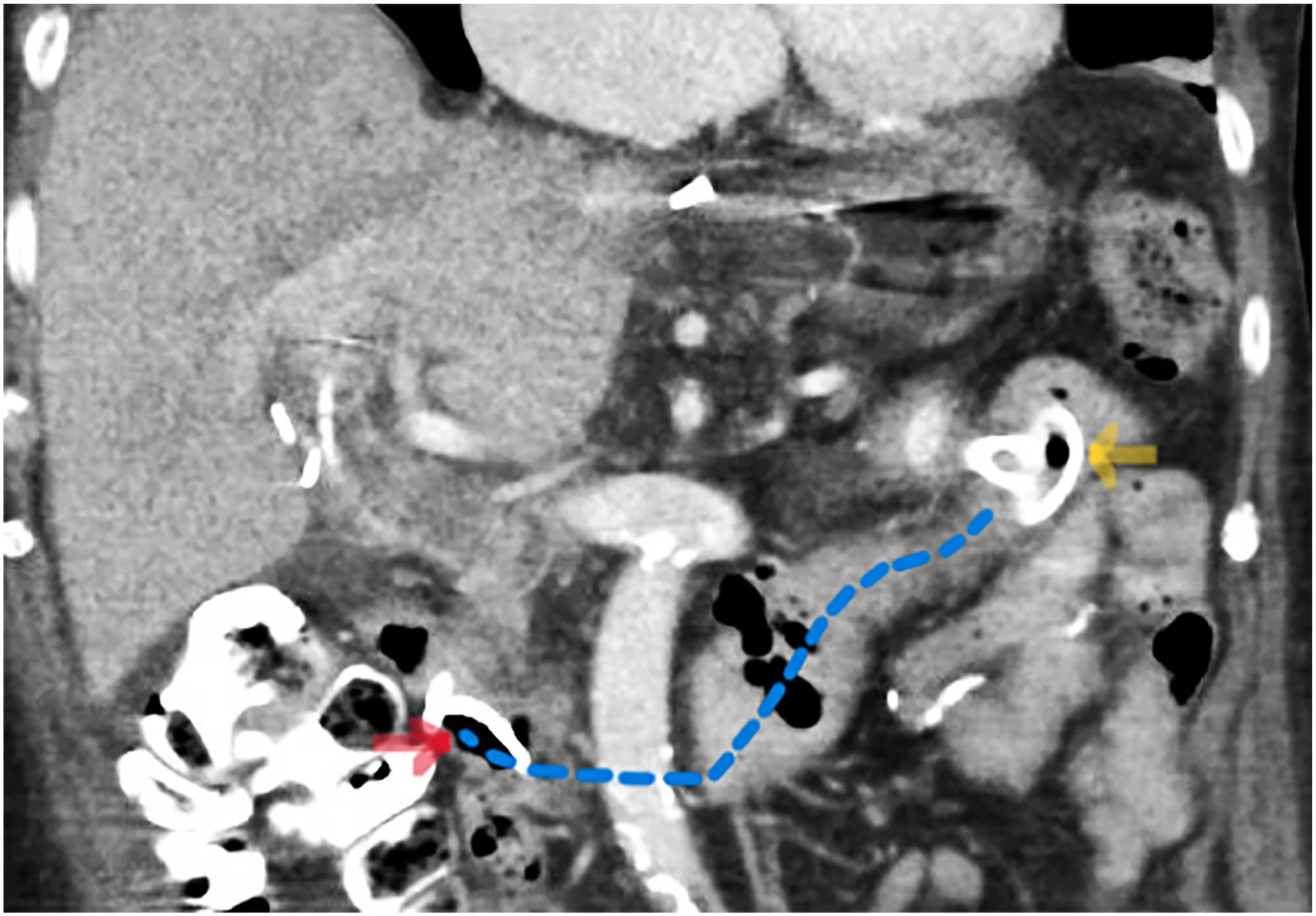
Patient 1: postoperative CT demonstrating the enteroenterostomy (*yellow arrow*), creating a short retrograde route (*blue dotted line*) to the papilla (*red arrow*).

Two weeks later, the PTBD was retracted after a wire was advanced into the duodenum. A regular gastroscope was advanced through LAMS in a retrograde fashion to the papilla. A guidewire was coiled in the duodenal bulb before the gastroscope was removed. Next, a duodenoscope (TJF-Q190V, Olympus) was advanced in a retrograde fashion under wire guidance to the papilla (resembling a Billroth II route). The bile duct was cannulated alongside the rendezvous wire, and a cholangiogram revealed large stones (Fig. 3). A partial sphincterotomy was followed by balloon sphincteroplasty (12 mm). A cholangioscope was then inserted, and large stones were fragmented with EHL (Autolith Touch, Boston Scientific). The fragments were removed with a balloon.

**Figure 3:**
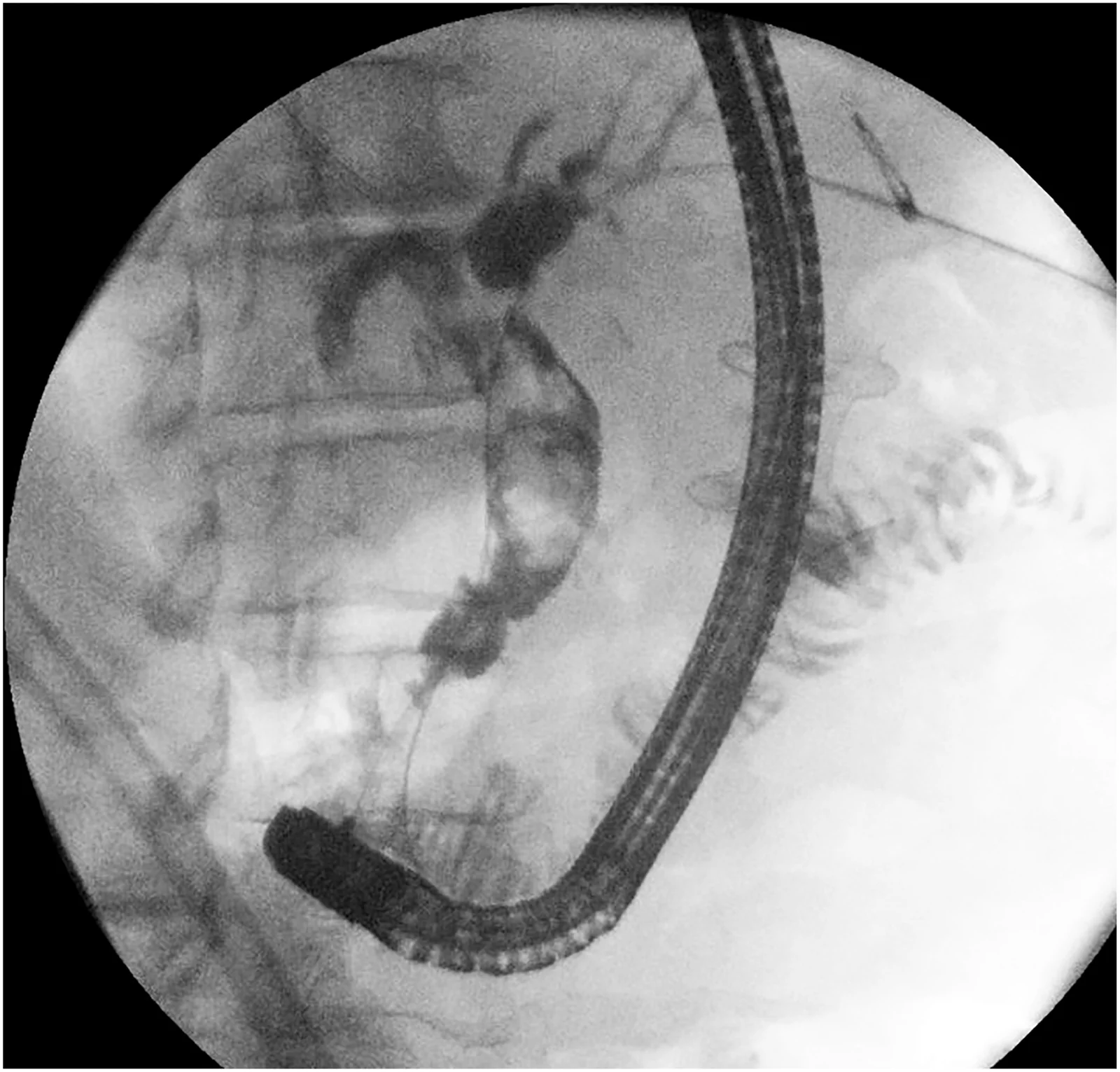
Patient 1: ERCP through lumen-apposing metal stent resembling a Billroth II ERCP.

Because of edema at the papillary orifice, a 10- × 6-cm fully covered self-expandable metal stent (FCSEMS) was deployed.

Four weeks later, cholangioscopy through the FCSEMS confirmed complete clearance of the bile duct. The FCSEMS was removed; a balloon sweep and final cholangiogram confirmed clearance. Subsequently, the patient's PTBD and LAMS were removed. The enteroenterostomy fistula was not closed.

The patient had transient episodes of atrial fibrillation with rapid ventricular response (American Society for Gastrointestinal Endoscopy lexicon for endoscopic adverse events: moderate) after the procedures that were treated medically. These episodes were attributed to underlying cardiac comorbidities and anesthesia rather than a direct effect of the endoscopic procedure. No other adverse events (AEs) occurred.

A year after her last ERCP, the patient remained asymptomatic during follow-up.

### Patient 2

A 77-year-old woman with a history of cholecystectomy and RYHJ performed abroad at age 23 (operative report unavailable) presented to an outside facility with cholangitis.

MRCP revealed biliary stones and HJ stricture. Bilateral PTBDs were placed after a failed enteroscopy-assisted ERCP. She underwent percutaneous cholangioscopy with EHL. The postoperative course was notable for a 2.9-cm extraluminal fluid collection, which was treated conservatively. Repeated MRCP showed persistent IHD dilation with residual stones. She experienced multiple PTBD problems, including migration, dislodgement, and bile leak at the skin; all were managed by drain replacements. She was referred to our institution for endoscopic internalization of her drains and stone extraction.

On CT, good apposition was observed between the duodenal bulb and the biliary limb near the HJ. After a multidisciplinary discussion, a decision was made to proceed with EDEE because of the AEs the patient experienced from the percutaneous biliary drain and the ease of reintervention with EDEE if needed.

A linear echoendoscope was advanced to the duodenal bulb. The biliary limb was identified next to the liver and punctured with a 19-gauge needle. Despite using an auxiliary pump, the target loop did not distend adequately. Our IR colleagues exchanged a PTBD for an 8F catheter without side holes. The catheter tip was positioned beyond the HJ. Contrast mixed with saline solution was instilled through the catheter using the pump, achieving adequate distention (Fig. 4). EC-LAMS (1.5 × 1 cm) was deployed, connecting the duodenal bulb to the biliary limb near the HJ (Fig. 5). Her PTBD was subsequently replaced.

**Figure 4:**
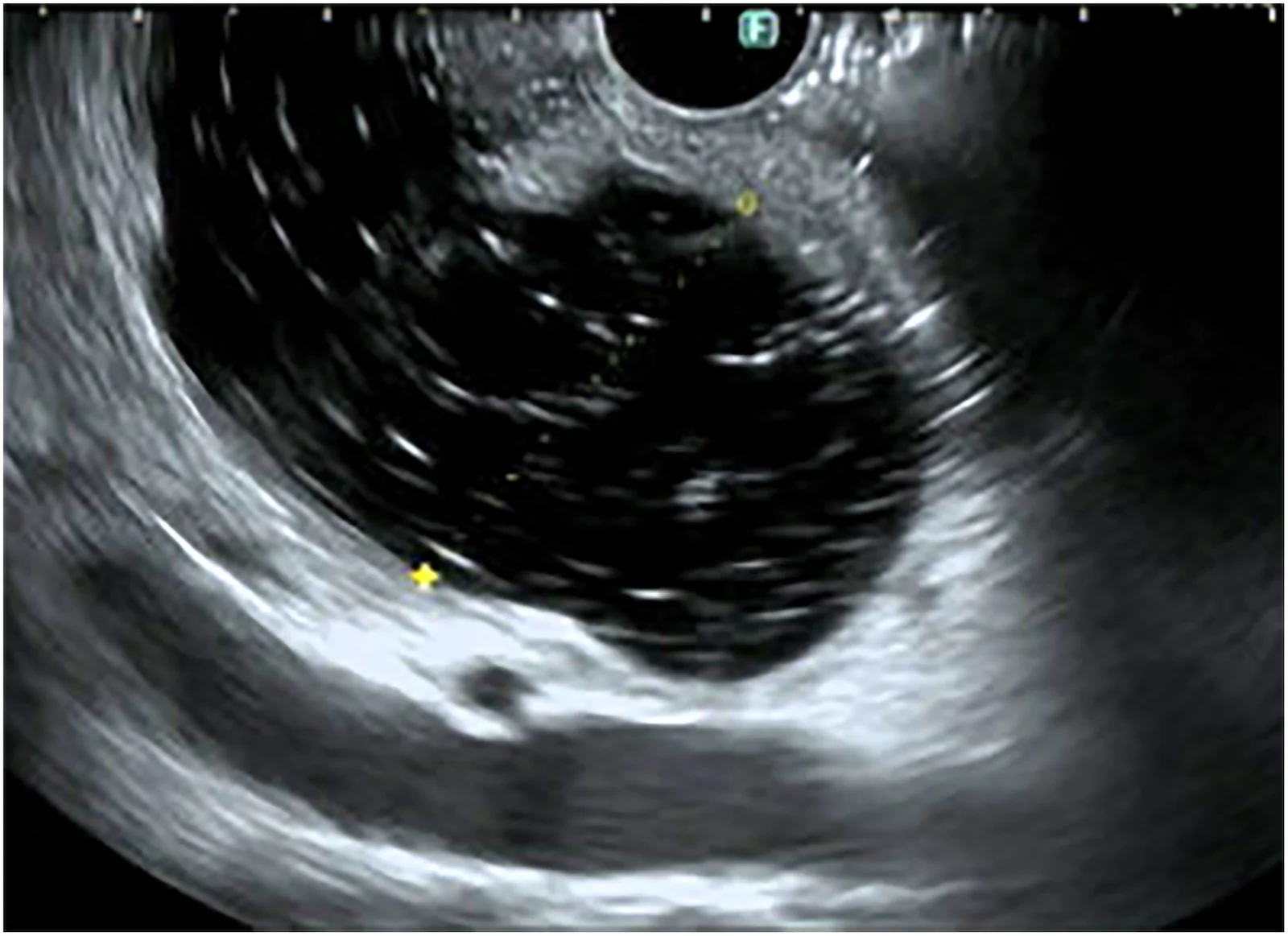
Patient 2: adequate distension of the targeted jejunal loop was achieved through the percutaneous access.

**Figure 5:**
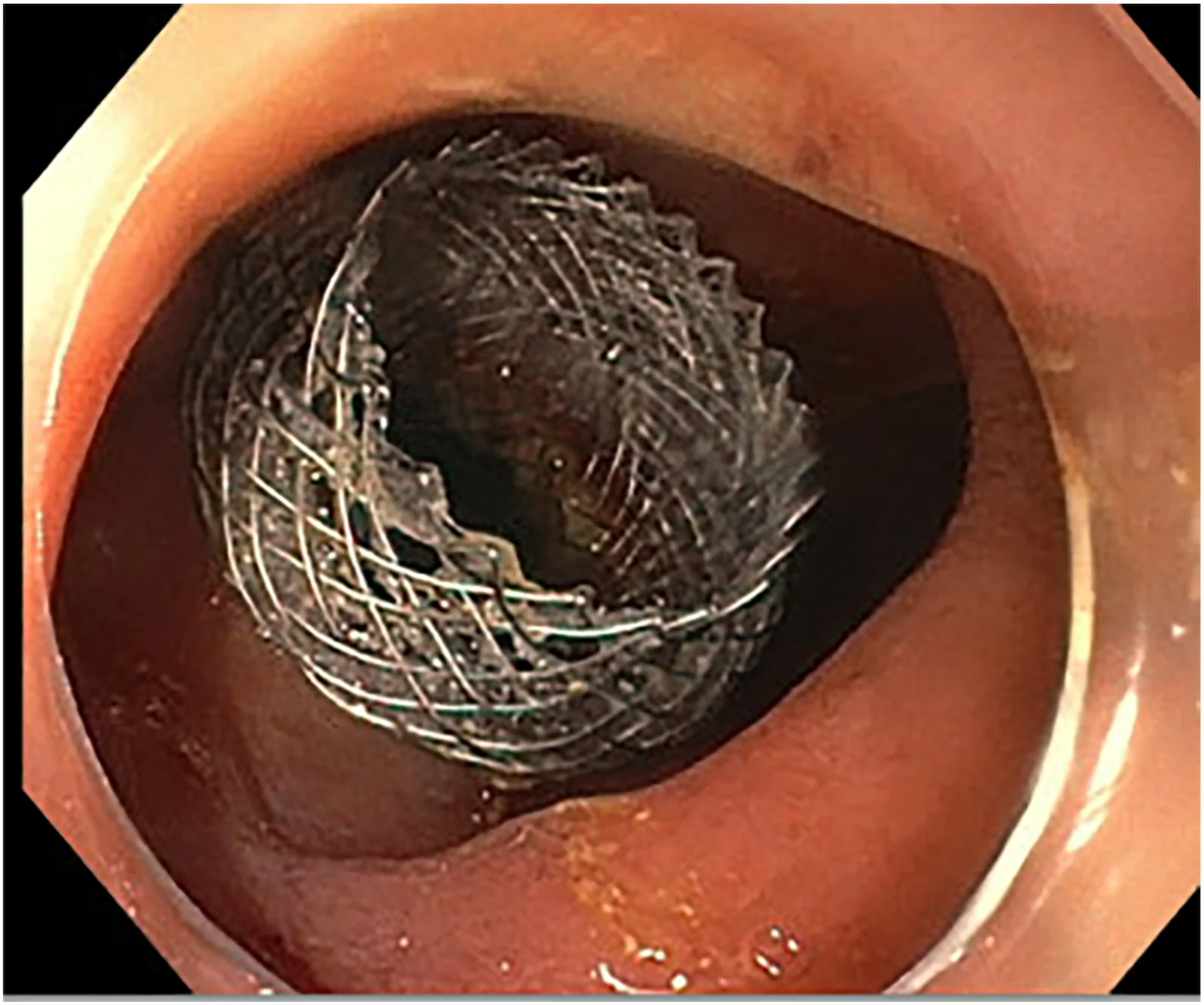
Patient 2: lumen-apposing metal stent after full deployment at the duodenojejunostomy.

Four weeks later, the LAMS was removed (it interfered with ERCP because of its proximity to the HJ), and a therapeutic gastroscope was advanced through the duodenojejunostomy to reach the HJ. The PTBDs were removed, and a cholangiogram revealed a dilated biliary tree with multiple filling defects (Fig. 6). The stricture was dilated with an 8-mm balloon, and multiple stones were removed using an extraction balloon. Subsequently, two 7F 3-cm double-pigtail stents were deployed, and a new LAMS (1.5 × 1 cm) was placed across the duodenojejunostomy.

**Figure 6:**
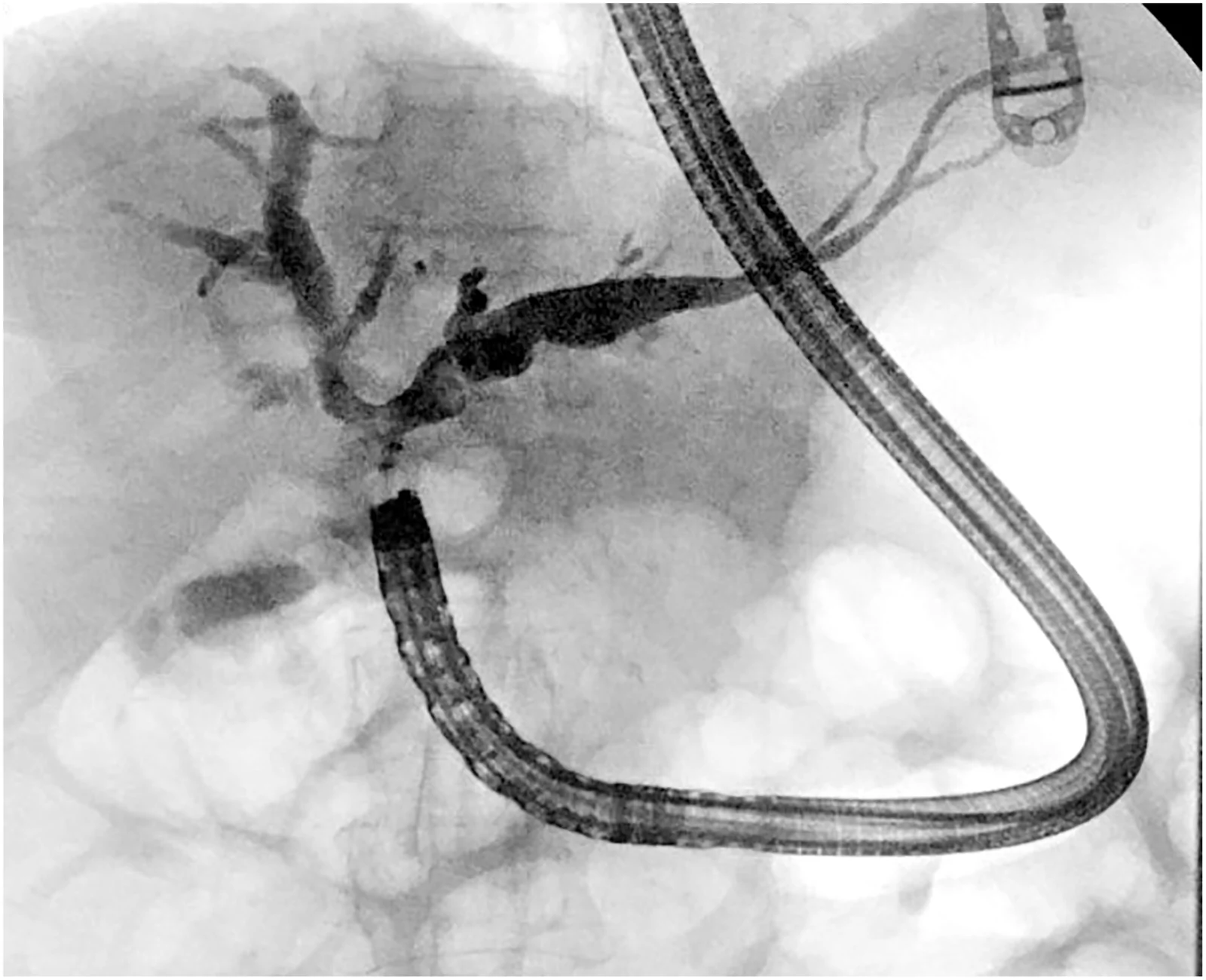
Patient 2: ERCP through the duodenojejunostomy.

The patient experienced temporary abdominal pain after her first procedure. Postoperative CT showed no AEs related to the procedure, and her pain resolved after treatment for underlying constipation (American Society for Gastrointestinal Endoscopy lexicon for endoscopic adverse events: mild).

Two months later, the LAMS and biliary stents were removed. The cholangiogram showed resolution of the stricture and no filling defects. Cholangioscopy confirmed that the bile ducts were clear of stones. At this stage, biliary stent placement was no longer necessary. We decided not to replace the LAMS to prevent food buildup at the HJ. Instead, two 7F, 3-cm double-pigtail stents were placed across the duodenojejunostomy.

### Patient 3

A 78-year-old woman with a history of early-stage cholangiocarcinoma who underwent surgical resection with RYHJ presented with cholangitis. On CT, good apposition was observed between the gastric antrum and the biliary limb. After a multidisciplinary discussion, a decision was made to proceed with EDEE because of suspicion of a stricture at the biliary anastomosis, which is often associated with multiple interventions.

The biliary limb was identified on EUS from the gastric antrum and punctured with a 19-gauge needle. After initial contrast injection confirmed the target by opacifying the biliary tree, the needle was connected to the auxiliary pump, and the biliary limb was further expanded after glucagon administration (Fig. 7). A 2- × 1-cm EC-LAMS was deployed, connecting the antrum to the biliary limb (Fig. 8). Because the patient was clinically stable on antibiotics, we elected to delay ERCP for 2 weeks to minimize the risk of LAMS dislodgement.

**Figure 7:**
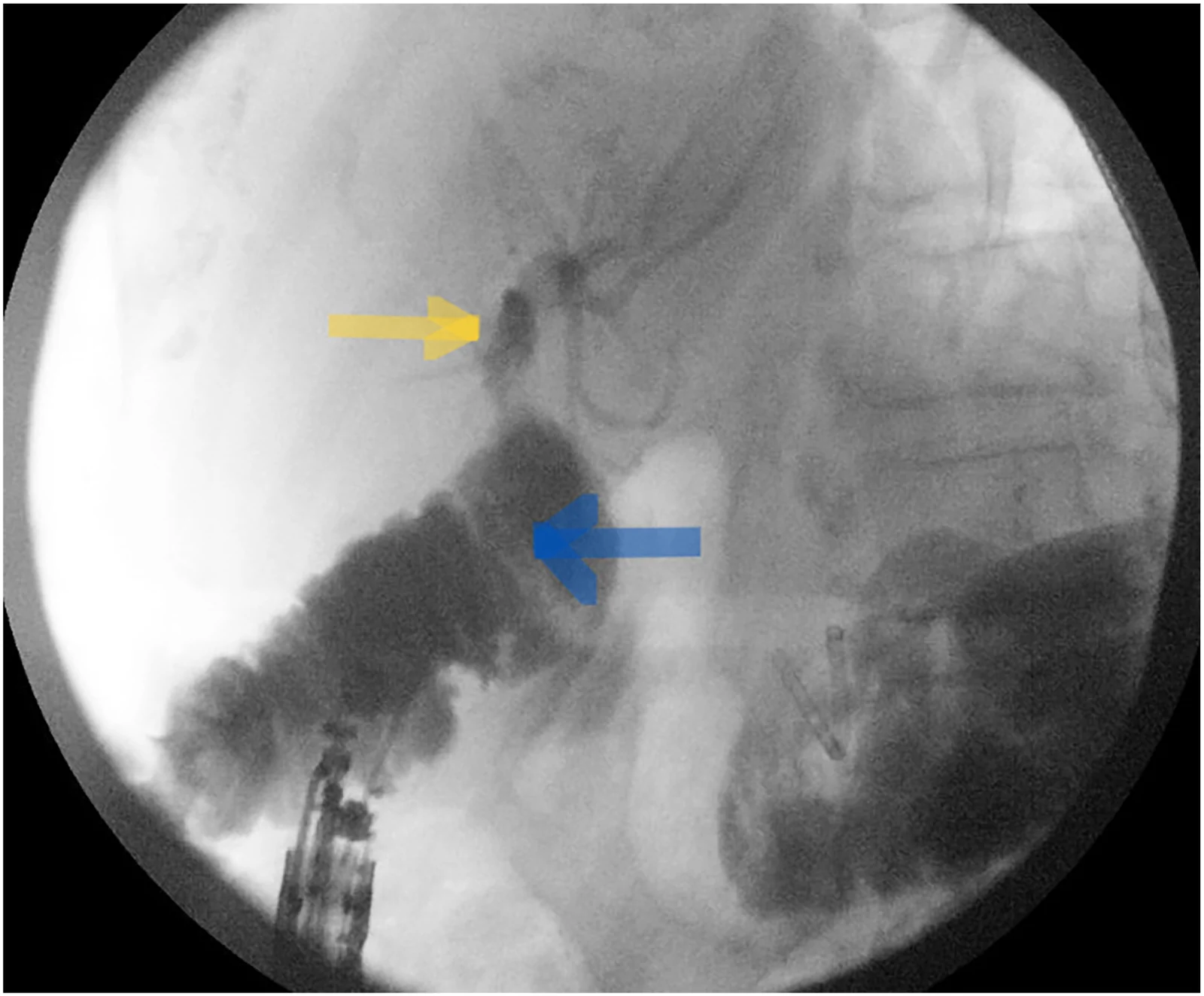
Patient 3: the target biliary limb (*blue arrow*) was distended using a direct needle puncture, resulting in opacification of the biliary tree (*yellow arrow*).

**Figure 8:**
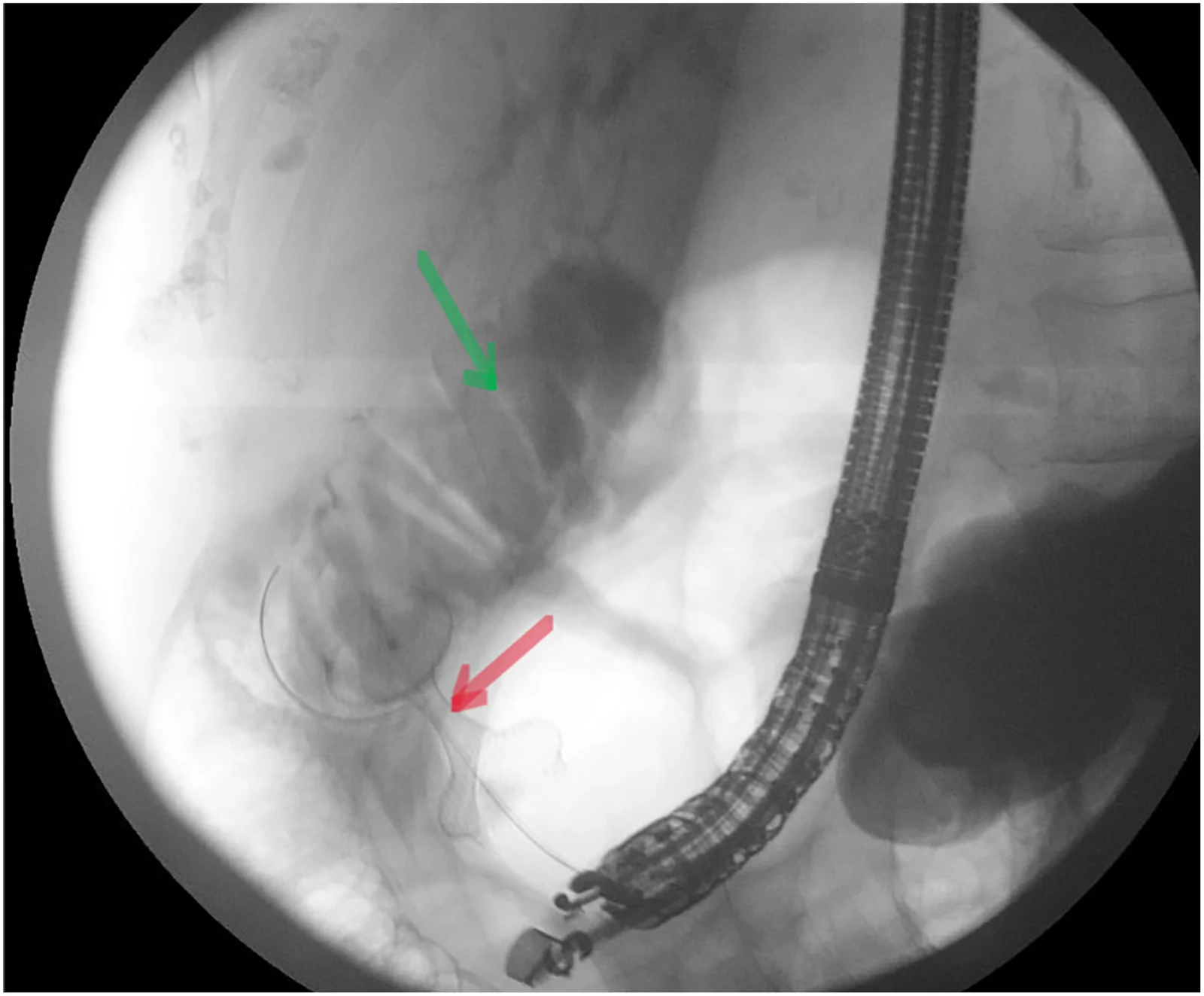
Patient 3: a fluoroscopic image of lumen-apposing metal stent (*red arrow*) connecting the gastric antrum to the biliary limb (*green arrow*).

Over the following 6 months, she underwent multiple ERCPs with serial dilations and stent placements (Fig. 9), ultimately achieving excellent biliary drainage. The LAMS was eventually removed without closing the gastroenteric fistula.

**Figure 9:**
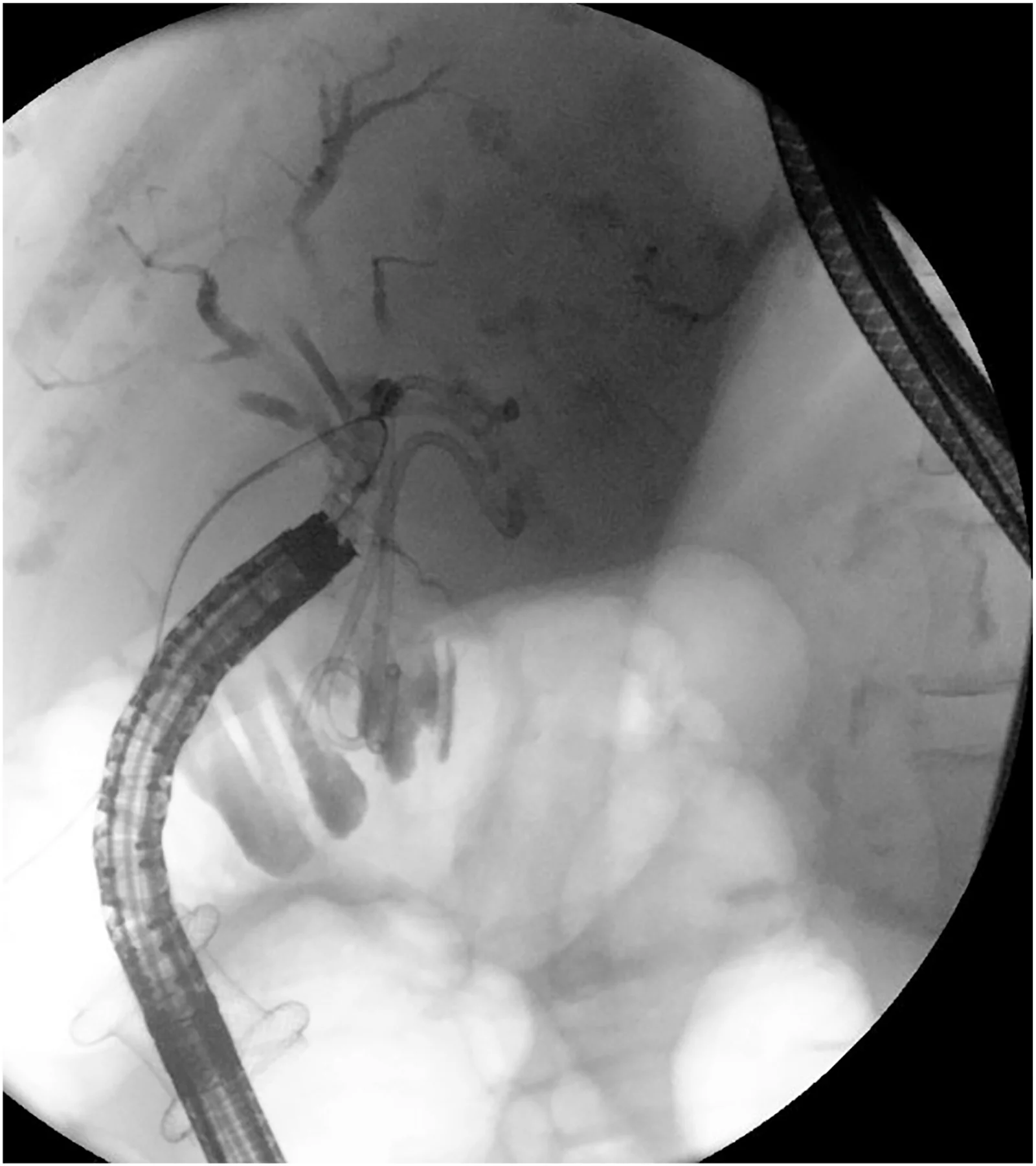
Patient 3: ERCP through lumen-apposing metal stent and biliary stent placement.

The patient experienced no AEs after the procedures. Her biliary stricture had been in remission, with no further biliary interventions over the past 3 years.

## Discussion

Our case series included 3 patients (2 with RYHJ and 1 with Roux-en-Y distal gastrectomy) who underwent EDEE to treat biliary strictures or stones or to replace the PTBD with endoscopic stent placement.

The feasibility of the procedure depends on identifying a small-bowel loop from the biliary limb that is well-opposed to the alimentary limb, without intervening organs or blood vessels.

Ideal indications for this technique include large biliary stones, >1 bilioenteric anastomosis, and conditions requiring multiple interventions. Contraindications include significant ascites, coagulopathy, and major comorbidities that prevent the use of anesthesia.

Our case series highlighted 2 technical variants of EDEE and provided tips for identifying and distending the target small-bowel loop. In the Roux-en-Y gastrectomy variant, the preferred target is the small-bowel loop adjacent to the ligament of Treitz. Conversely, for the RYHJ variant, the optimal—although not always feasible—target is the biliary limb near the HJ (as in patient 3), rather than directly at the anastomosis (as in patient 2). Targeting the anastomosis itself may increase the risk of food accumulation at the bilioenteric junction and may require removal of the LAMS during ERCP, because the stent flange can interfere with the procedure, thereby increasing procedural costs.

In our case series, all procedures were performed as 2-step procedures because of the lack of urgency and the risk of stent dislodgement associated with 1-step procedures.

Although LAMS fixation has been proposed to reduce migration risk in 1-step procedures, there are currently no data supporting its use in EDEE procedures. In theory, fixing the proximal flange does not prevent dislodgement of the distal flange if enough tension is applied at the anastomosis. In the largest retrospective study on EDEE by Pérez-Cuadrado-Robles et al,^9^ which included 55 patients, LAMS fixation (performed in one-third of patients) was not associated with fewer LAMS-related AEs.

In general, LAMS dislodgment during EDEE is related to the angulation at the entry to the biliary limb and the type of endoscope used. Although standard forward-viewing gastroscopes are the safest and easiest to pass, they can only accommodate 7F stents. This point should be considered before deciding between a 1-step and a staged EDEE based on the procedure's indication.

All procedures in our case series were both technically and clinically successful. EDEE provided easy access to the biliary orifice, enabling straightforward, repeatable ERCPs. One case involved a patient with a Roux-en-Y distal gastrectomy and large impacted stones, a challenging scenario even with normal anatomy. By creating a short route (similar to Billroth II), EDEE facilitated the use of a dedicated duodenoscope and a cholangioscope with EHL, allowing efficient, complete bile duct clearance.

We do not routinely close the transmural fistula after LAMS removal unless it causes adverse effects, such as food buildup at the HJ (if the fistula was too close to the bilioenteric anastomosis). Other AEs associated with EDEE include bleeding, perforation from injury to the opposite wall of the jejunum or from LAMS maldeployment or dislodgment (if the tract is not mature), and peritonitis.

There were no procedure-specific AEs in our case series.

This procedure requires a high level of experience and a multidisciplinary team approach with surgical and IR backup.

## Conclusions

At experienced centers, EDEE is a valuable technique for biliary interventions in patients with nongastric bypass Roux-en-Y reconstruction. By creating a shorter route and enabling the use of specialized tools, it facilitates both access to the biliary orifice and the execution of the biliary procedure. In addition, it enables faster, simpler repeat interventions compared with balloon-assisted ERCP.

Further data are needed to assess the safety and long-term effectiveness of this technique compared with other endoscopic approaches.

## Disclosure

All authors disclosed no financial relationships.

## Supplementary data

Video 1 EUS-directed transenteric ERCPfor biliary interventions in non-gastric bypass Roux-en-Y reconstruction.
